# Sex differences and considerations for female specific nutritional strategies: a narrative review

**DOI:** 10.1186/s12970-021-00422-8

**Published:** 2021-04-01

**Authors:** Kealey J. Wohlgemuth, Luke R. Arieta, Gabrielle J. Brewer, Andrew L. Hoselton, Lacey M. Gould, Abbie E. Smith-Ryan

**Affiliations:** 1 Department of Exercise and Sport Science, University of North Carolina at Chapel Hill, Chapel Hill, NC 27599 USA; 2 Department of Nutrition, Gillings School of Public Health, University of North Carolina, Chapel Hill, NC 27599 USA

**Keywords:** Women’s health, Human performance, Menstrual cycle, Dietary supplement, Female, Macronutrients

## Abstract

Although there is a plethora of information available regarding the impact of nutrition on exercise performance, many recommendations are based on male needs due to the dominance of male participation in the nutrition and exercise science literature. Female participation in sport and exercise is prevalent, making it vital for guidelines to address the sex-specific nutritional needs. Female hormonal levels, such as estrogen and progesterone, fluctuate throughout the mensural cycle and lifecycle requiring more attention for effective nutritional considerations. Sex-specific nutritional recommendations and guidelines for the active female and female athlete have been lacking to date and warrant further consideration. This review provides a practical overview of key physiological and nutritional considerations for the active female. Available literature regarding sex-specific nutrition and dietary supplement guidelines for women has been synthesized, offering evidenced-based practical information that can be incorporated into the daily lives of women to improve performance, body composition, and overall health.

## Introduction

Now, more than ever, there is a high prevalence of female participation in physical activity and sport [1, 2]. There is also a growing awareness of the potential impact of cyclical menstrual hormones (i.e. estrogen and progesterone) on exercise performance [3] and metabolic demand [4], making it important to understand physiological differences and address female specific nutritional needs [5]. Over the course of the menstrual cycle, it is important to note that women may have different caloric and macronutrient needs due to fluctuations in sex hormones, substrate reliance, and increased energy demand during exercise [6, 7]. Macronutrient ranges vary per individual depending on menstrual cycle, energy demands, and/or body composition goals [8–10]. These ranges should be primarily met with whole foods; however, key dietary supplements may be beneficial to females to support improvements in performance, recovery, and overall health [11]. In addition to physiological sex differences, there are notable reported behavioral differences between males and females, such as males wanting to be larger with more muscle mass, while females tend to want to be leaner and have reported more attempts to lose weight [12, 13]. It is also known that most female athletes under consume calories [14, 15]; however, it is vital for overall health and performance that women meet their caloric needs to maintain energy availability and regular menstruation [16, 17]. To date, there are few resources that provide a physiological rationale for sex-specific nutritional needs and dietary guidelines for women, particularly in a single resource. This review aims to provide an evidence-based, but practical overview, for nutrition and dietary supplements in healthy eumenorrheic women.

## Physiological sex differences

**Fig. 1:**
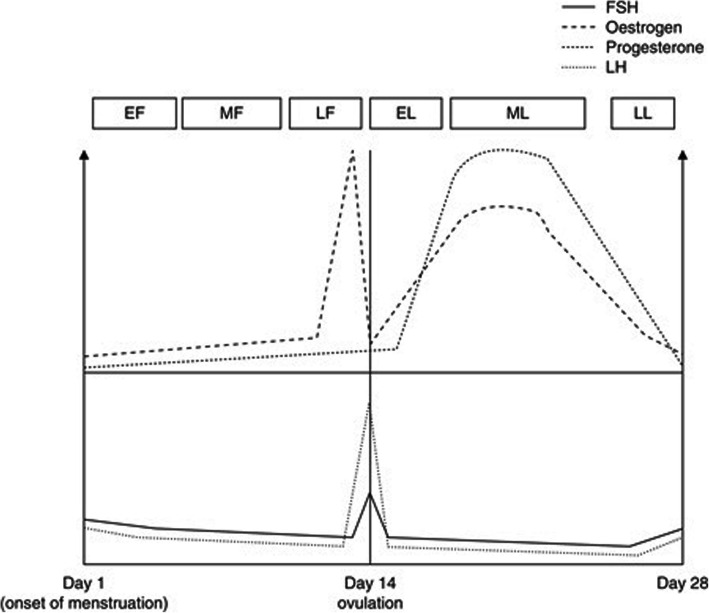
Female sex hormones fluctuate cyclically and predictably throughout the menstrual cycle. Estradiol is the primary estrogen secreted, and it is depicted as estrogen in this figure. The phases are defined as follows: EF = early follicular; EL = early luteal; FSH = follicle stimulating hormone; LF = late follicular; LL = late luteal; MF = mid follicular; ML = mid luteal. This diagram was adapted from Oosthuyse & Bosch (2010) [25]

Understanding physiological sex-based differences between men and women may help optimize nutritional strategies chosen to support certain goals ranging from maximizing exercise performance to gaining lean mass or losing weight. Important sex-differences exist in substrate utilization [18], thermoregulation [19], fatigability [20], soreness and recovery [21], and body composition [22]. Prior to puberty, males and females demonstrate similar substrate metabolism [23]; in adulthood estrogen has a large regulatory role in fat metabolism [24]. Once completing the transition through puberty, if caloric intake is adequate, women typically experience regular menstrual cycles, predictable fluctuations of ovarian hormones, and are considered eumenorrheic until they begin the menopause transition (average age of onset is 51 years). A regular menstrual cycle lasts an average of 28 days (ranging from 21 to 45 days) and consists of two main phases: the follicular phase and the luteal phase (Fig. 1) [25]. At the beginning of the follicular phase (~ 14 days), menstruation occurs and hormones, including estrogen, specifically 17β-oestradiol (estradiol), progesterone, follicle stimulating hormone (FSH), and luteinizing hormone (LH) are at their lowest baseline levels [25, 26]. Around day 4–5 of menstruation, the mid-follicular phase begins, and estrogen and FSH levels rise to prepare the body for ovulation. At the end of the follicular phase (day 11–13), LH spikes and induces ovulation, while estrogen rises and then falls following ovulation. The luteal phase (14 days) begins after ovulation occurs; LH and FSH decline and return to baseline levels while progesterone and estrogen levels peak around day 20–24 (mid-luteal phase) to prepare the body for pregnancy. If pregnancy does not occur, hormone levels return to baseline and induce menstruation, signaling the beginning of a new menstrual cycle [27].

**Fig. 2:**
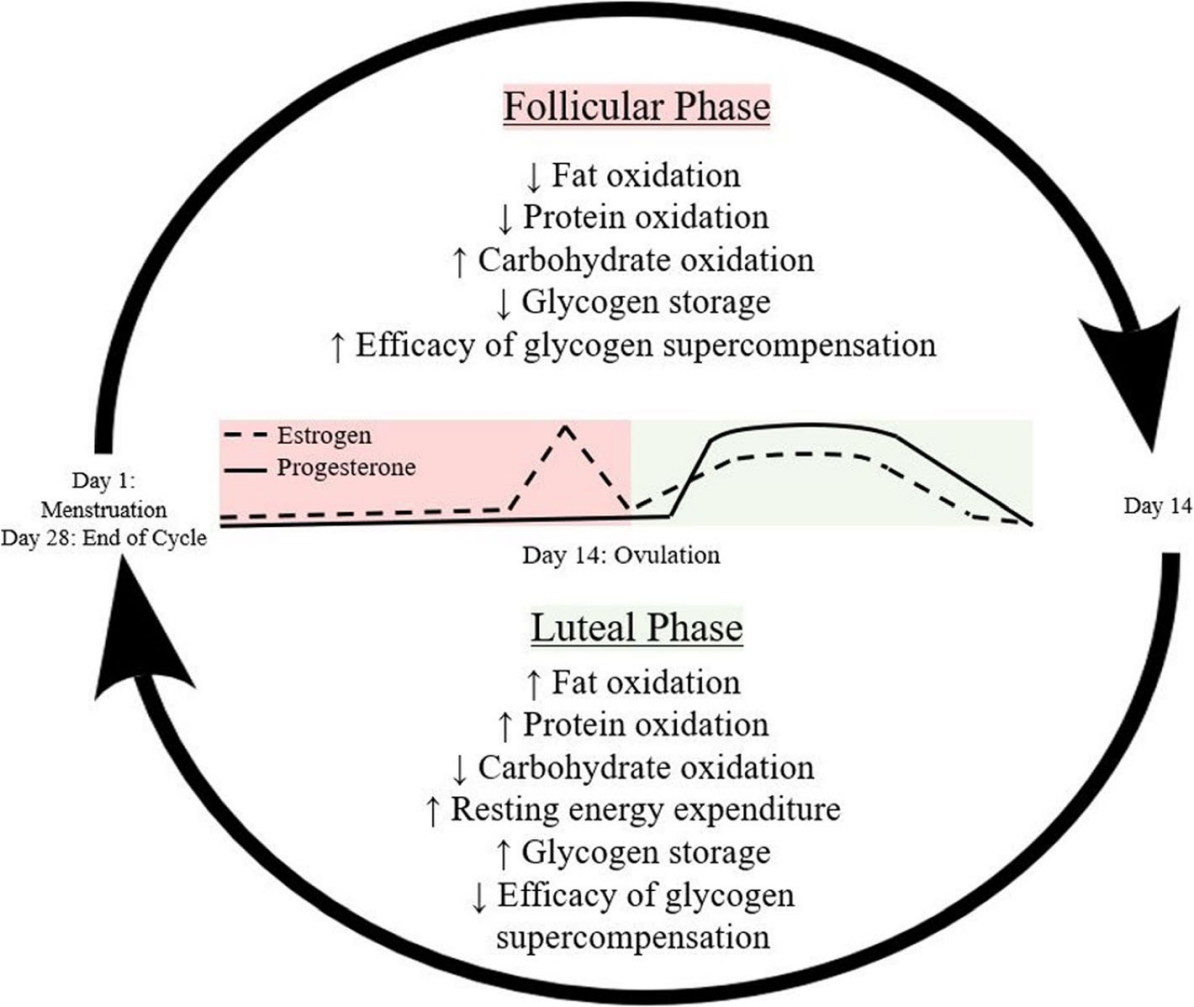
Nutritional needs throughout the menstrual cycle may change based on physiological implications from estrogen and progesterone. Key metabolic adaptations are described for the follicular and luteal phases

It is important to consider the effects of the menstrual cycle on metabolism and performance as women exercise and compete in every phase of their cycle, and may be able to optimize nutritional strategies based on these influences. Substantial evidence suggests that estrogen is a master regulator of both body composition and bioenergetics, so the fluctuation of estradiol throughout the menstrual cycle may have important implications for exercise capacity and nutrition [28]. At rest, women exhibit heightened fat oxidation, as indicated by decreased respiratory exchange ratio (RER), and 2.5–11.5% higher resting energy expenditure (REE) during the luteal phase of the menstrual cycle when ovarian hormones peak [24, 29, 30]. However, consistent exercise training may also dictate substrate utilization and improve fat oxidation efficiency in men, which is supported by insignificant differences in RER between endurance-trained males and females [6]. While studies that utilize indirect calorimetry have provided substantial evidence for increased fat oxidation at rest in women, this methodology is unable to quantify protein catabolism. Through measuring circulating amino acids in the bloodstream and urine, studies have found that protein oxidation is also greater at rest in women during the luteal phase [31, 32]. Taken together, these findings indicate that increased metabolism of fat and protein occurs during the luteal phase, which is accompanied by greater caloric expenditure, and possibly appetite. Figure 2 depicts the impact of menstrual cycle phase on metabolism and performance [25].

### Aerobic exercise

Because sex hormones like estradiol may influence metabolism and other aspects of physiology at rest and during exercise, it is important to consider how sex hormones affect exercise performance. Understanding when performance may be naturally augmented or impeded by the menstrual cycle may allow women to optimize their nutrition. Evidence suggests that increased estradiol in the luteal phase spares muscle glycogen by promoting oxidation of free fatty acids (FFA), which would have implications for improved endurance performance [25]. During submaximal exercise of the same intensity, adult women generally metabolize more fat compared to men, which is indicated by a lower RER [33, 34]. A more recent review details that women may confer an advantage in ultra-endurance competition [35]. Although research assessing prolonged-duration exercise in women is scarce, it is hypothesized that ultra-distance (> 2 h) performance may be impaired due to the suppression of gluconeogenic output when estrogen and progesterone are at high levels (i.e. luteal phase) [36]. One study assessed time to exhaustion (TTE), a proxy for endurance performance, following glycogen depletion exercise during their follicular and luteal phases, and found that exercise in the luteal phase produced a 10% longer TTE and was associated with greater glycogen repletion [37]. A different study found that time trial performance was 13% better in the follicular phase following 120 min of cycling at 70% maximal oxygen consumption (VO_2_max), indicating that intense exercise may be supported better by increased glucose kinetics in the follicular phase [7]. While it is not feasible to modify competition dates around the menstrual cycle, based on this data, it may be beneficial to increase carbohydrate consumption to support higher glycogen levels, during the luteal phase. Ovarian hormones have generally been found ineffective in altering short-duration or maximal effort exercise, however, one study found that VO_2_max was 2% lower in the mid-luteal phase [38–40]. The effects of oral contraceptives on performance is unclear; however one study demonstrated that VO_2_max was reduced by 11% in women who consumed oral contraceptives for 4 months [41]; these effects are likely related to the type of contraception.

### Strength & Power

To date, most literature suggests that the menstrual cycle does not directly influence strength or power exercise performance. One study tested half-squat 1 repetition maximum (1RM) in resistance-trained women during their early follicular, late follicular, and mid-luteal phases; this group measured force production, velocity, and power at loads equivalent to 20, 40, 60, and 80% of their 1RM and found similar results across all three menstrual phases [42]. Taken together, the menstrual cycle likely impacts endurance performance, but not resistance performance, by altering substrate utilization, which may be further impacted by the ratio of the magnitude of increase in estrogen and progesterone in the luteal phase [25]. Indirectly, other aspects of the menstrual cycle may influence both endurance and strength/power activities. Specifically, sex differences in fatiguability, thermoregulation, and body composition should be considered.

### Fatiguability

A recent review reports that fatigability differs between men and women, with women being less fatigable during single-limb isometric contractions [20]. Women proportionally have greater area of type I muscle fibers in numerous muscle groups, which is a proposed mechanism to support reduced fatigability in women during isometric contractions [43]. Another study found similar results regarding dynamic contractions, when the load was low and contractions were slow-velocity, women were less susceptible to fatigue [44]. Collectively, available data suggest that men and women may respond differently to training that involves fatiguing contractions and may require altered recovery and nutritional support. While women appear to be less fatigable during exercise, it is also important to consider sex-differences in the recovery period following exercise. Hormonal fluctuations from the menstrual cycle have been shown to alter recovery rates in women. A recent study in 8 trained, eumenorrheic females reported that markers of muscle damage (creatine kinase) and inflammation (interleukin-6) following 90 min of running at 70% VO_2_max were substantially greater during the follicular phase compared to the mid-luteal phase [21]. Thus, nutrition and exercise recommendations should consider phase of the menstrual cycle to maximize performance and recovery. Specifically, recovery rates may be impeded in the follicular phase. Additionally, females report greater blood flow and lower metabolic acidosis, particularly during intermittent exercise [45]; consequently, higher intensities and shorter rest periods, as well as variation in dietary supplements that delay fatigue, may need a sex-specific lens.

### Thermoregulation

Thermoregulation may vary between sexes; during heat exposure, the onset of sweating was delayed and resulted in lower volume in women compared to men [46, 47]. Interestingly, when men and women are matched for age, acclimatization, body size, and fitness level, differences in thermoregulation no longer persist, however, many studies fail to control for the menstrual cycle in females [48]. Issues related to thermoregulation likely impact women to a greater extent than men because the luteal phase of the menstrual cycle is associated with higher core body temperature, greater cardiovascular strain during submaximal steady-state exercise, and a heightened threshold for the onset of sweating [19, 49, 50]. It may be important to increase fluid intake during the luteal phase, as well as evaluate sweat rates during the various phases of the cycle to identify a woman’s fluid requirements and alterations.

### Body composition

In regard to body composition, it is important to remember that there are innate sex differences. Men generally have more lean mass (LM), while women have more fat mass (FM) and higher body fat percentage (%fat); men are more susceptible to gaining abdominal fat (android), whereas women usually carry more fat in the hip (gynoid) region [22]. Men and women may also respond differently to training and diet interventions, in regard to body mass and composition. A 24-week diet or diet plus exercise intervention was shown to significantly decrease body mass at three months (− 7.4 ± 0.5% for diet and − 5.8 ± 0.3% for diet plus exercise) and at six months (− 10.4 ± 0.9% for diet and − 10.1 ± 0.9% for diet plus exercise) compared to consuming a weight maintenance diet, but there was no effect for sex [51]. Similarly, there was no effect for sex on FM deduction; however, men lost a significantly greater amount of visceral adipose tissue in both intervention groups compared to women. No sex differences were seen in subcutaneous adipose tissue. Fat free mass (FFM) was reduced from baseline in all three groups at both three and six months for women, while men in the diet plus exercise had no significant reduction in FFM from baseline at three months and no reduction at three or six months for those consuming maintenance calories. A study assessing a three-week training and diet intervention on age and BMI matched obese males and females found that body mass was significantly reduced in both groups, but that men lost a greater percentage of their initial body mass [52]. Perhaps more importantly, men lost both FM and LM in similar amounts whereas women lost mostly FM. More recent data has suggested women are more resilient in maintaining LM under caloric restriction [53]. These distinct differences may be especially important with advancing age due to their influence on functional performance. Leg lean mass was shown to be associated with balance in older women in addition to a greater weight to leg lean mass ratio being predictive of performance during gait tasks, but neither relationship exists in older men [54]. Lastly, bone mineral density (BMD) can be evaluated to measure risk or current levels of osteoporosis or osteopenia by assessing bone mass [55]. Previous literature has reported that BMD decreases in both sexes with age; however, in adult to elderly women BMD is significantly lower than men, which may lead to increased osteoporotic fractures, so training and nutrition in women should be optimized to prevent loss of BMD [55].

## Overview of types of birth control on hormones

There are numerous contraception options available that can alter hormone fluctuations, including both hormonal and non-hormonal options. At the minimum, it would be important to identify what type of contraception a female is using, in order to better understand the potential implications, it may have on hormonal fluctuations, if any, and thus help guide nutritional recommendations. Only hormonal contraceptives will be discussed here, and in particular their influence on exercise rather than discussing their efficacy in regard to preventing pregnancy or their potential non-contraceptive benefits, such as decreasing ovarian cancer risk [56]. Hormonal contraceptives are available to be taken orally (a pill), as a patch for transdermal administration, as a vaginal ring, along with implants (e.g. in the upper arm) and intramuscular injections [57]. A data brief from the National Center for Health Statistics [58] estimated that 64.9% of all women aged 15–49 years in the United States are using some form of contraception with the oral contraceptive pill (OC) reported as the most common form of exogenous hormonal contraceptive. In general, OC consists of an estrogen (e.g. ethinyl estradiol or mestranol) and a progestin (e.g. norethindrone, norethindrone acetate, ethynodiol diacetate, norgestrel, or norethynodrel) [59]. To imitate the rising and falling of hormones over the course of the menstrual cycle, OC have phases [60]. Monophasic OC consist of the same estrogen and progestin levels throughout the cycle, while biphasic OC will maintain the same hormone levels for the first half of the cycle; however, progestin levels will increase halfway through the cycle, and triphasic pills have variations in hormones every 7 days [60, 61]. Different doses and types of progestin taken have been shown to have negative effects on plasma glucose, insulin, and C-peptide response to a glucose tolerance test [62]. Although, low doses of seven different types or concentrations of progestogen resulted in no change in glucose metabolism over six months [63]. During exercise, triphasic OC have been shown to decrease glucose flux and may reduce insulin action [64]. Lipid profiles may also be altered in women taking OC as a recent meta-analysis concluded that most progestins increase the concentrations of both triglycerides and high-density lipoprotein cholesterol, but effects on low-density lipoprotein cholesterol varied [65]. However, some have shown no difference in substrate utilization or hormonal response to 45-min of exercise at 65% VO_2_max in eumenorrheic women compared to women taking low-dose oral contraceptives [24], while others have shown increased glycerol appearance, indicative of triglyceride mobilization, and increased cortisol during exercise in women taking OC [66]. It should be noted that the use of a monophasic and triphasic OC, respectively, could potentially explain the differences seen. Oral contraceptive pills may have a negative influence on inflammatory markers as evidenced in elite Olympic level female athletes who were using oral contraceptives as they displayed higher levels of C-reactive protein, a marker of inflammation, than eumenorrheic peers, potentially suggesting increased muscle damage or inadequate recovery [67]. A study assessing less elite but still highly active females found similar results as those taking OC had higher levels of high-sensitivity C-reactive protein than non-OC users [68]. Further, there was no difference in cycling endurance performance [69] or swimming performance [70] in trained females taking a monophasic OC throughout a single OC cycle. However, triphasic OC have been shown to result in decreased aerobic capacity compared to no OC use [41, 71]. There was no difference in leg extensor isometric maximal muscular strength or 1-repetition maximum between OC users and non-users following 10-weeks of progressive lower body resistance training [72]. Interestingly, there was a trend (*P* = 0.06) towards a greater increase in muscle cross-sectional area in those taking OC, and this trend was driven by a greater increase in CSA from those taking OC with 30 μg compared to 20 μg ethinyl estradiol. Due to the plethora of females who use contraceptives, it is important to understand the different types available and the influence they may have on the body, specifically, on metabolism and performance. While some physiological variables may be altered during exercise with hormonal contraceptive use, careful attention should be paid to the type of and concentration of the exogenous hormones as these may elicit distinct responses.

## Caloric intake considerations

A primary nutrition consideration for women should be achieving adequate caloric intake, particularly with increased levels of physical activity, in order to support cellular function and performance demands. Previous data evaluating dietary intake and eating habits of female athletes reported that the majority of athletes under consumed calories, in particular carbohydrates [14]. Failure to reach sufficient metabolic and caloric demands may elicit disruptions to menstruation, performance, and bone mass, potentially increasing the risk for injury and osteoporosis [17, 73].

Energy availability (EA) is the remaining dietary energy in the body after the energy expenditure from physical activity has been considered [74]; this remaining dietary energy is available to be utilized in the body’s metabolic processes [75]. Failure to habitually maintain adequate caloric intake necessary for metabolism may result in health consequences and performance determinants, which is particularly prevalent among women [14]. These consequences may be further exacerbated with the addition of physical activity, particularly with increased intensity and duration of exercise. Previous data reported that 5 consecutive days of low EA (< 30 kcal/kg fat-free mass/day) in women resulted in a reduction of carbohydrate availability [74], which would have direct implications on exercise performance.

Obtaining optimal EA (40–45 kcal/kg FFM/day), eumenorrhea, and bone health are essential to sustaining metabolism and maximizing performance in women [76]. Lack of proper nutrition may lead to Relative Energy Deficiency in Sport (RED-S)/Female Athlete Triad (Triad) [77]. RED-S and the Triad both refer to disordered eating, or under consumption, and osteoporosis; the Triad includes amenorrhea as an additional consideration for women. In the absence of a menstrual cycle, Disordered eating often leads to energy imbalances and deficits which can result in amenorrhea, decreased performance, and in severe cases death. Amenorrhea, or the absence of a menstrual cycle, would result in different nutritional recommendations as discussed here due to the lack of cyclical hormone variation. Specifically, amenorrhea is characterized by a lack of ovulation as a result of insufficient luteinizing hormone and follicular stimulating hormone; both of which would impact estrogen and progesterone concentrations. For specific nutritional recommendations for amenorrhoeic athletes see Benson et al. [78]. Osteoporosis is defined as a bone mineral density (BMD) below 2.5 standard deviations of a young healthy adult and leads to porosity of the bones increasing the chances of fracture [79]. While these conditions can occur independently, low BMD is often an indicator of menstrual disturbances in younger women [79]. Combined, the effects of RED-S or the Triad behaviors influence metabolic function, muscle protein synthesis, bone health, immunity, and cardiovascular health; all of which may alter daily living and both short- and long-term performance [80]. Best practices to avoid RED-S or the Triad include adequate caloric intake to meet energy, metabolic, and activity demands and sufficient intake of proper macronutrients. In addition to achieving optimal EA, it may be advantageous to increase caloric intake and certain macronutrients throughout certain phases of the menstrual cycle. Previous data regarding REE through the cycle suggests day-to-day variability, with some women experiencing an increase up to 2.5–11.5% during the luteal phase [29, 81]. Once the caloric needs have been met, addressing nutrient timing of carbohydrates, fats, and protein may be advantageous to further optimize performance and recovery.

## Carbohydrates

Carbohydrates serve as a vital fuel source during moderate to high intensity exercise [82]. Currently, the acceptable macronutrient distribution range (AMDR) for carbohydrates is 45–65% of total calories [82], with additional recommendations of 6–10 g/kg/day in active populations looking to maximize performance [83]. Ultimately, carbohydrate needs are largely dependent upon the length and intensity of training or competition, with longer more intense activities exacerbating the demand [84]. Strategic implementation of carbohydrates around an exercise bout is essential to ensuring carbohydrate availability [85]. Sex differences and the variations in sex hormones over the menstrual cycle also influence the utilization and storage of carbohydrates [86] and must be considered.

The correlation between endurance exercise performance and pre-exercise muscle glycogen content [87] suggests that carbohydrate loading programs can increase performance [86]. With most of the carbohydrate loading studies involving men, it was assumed that that similar guidelines would be applicable to females [88]. However, previous research shows that men and women differ in the ability to carbohydrate load following the same protocol. Tarnopolsky et al. [89] implemented a feeding protocol that increased daily carbohydrate intake from 55 to 60 to 70% energy intake in trained male and female cyclists over a 4-day tapering period. The females, who were in the follicular phase of the menstrual cycle exhibited no significant changes in muscle glycogen content or performance on a submaximal endurance test, while males improved on both parameters (41 and 45%, respectively). Although the relative carbohydrate intake was equal among the sexes, the elevated overall energy intake in males resulted in greater absolute carbohydrate intake. Thus, for females, emphasis must be placed on adhering to g/kg recommendations. Walker and colleagues [9], further investigated the effects of carbohydrate loading programs in trained women during the luteal phase of the menstrual cycle. A high carbohydrate diet (8.2 g CHO/kg BM/day) resulted in significantly higher muscle glycogen content (13%) and improved performance on a submaximal endurance test compared to moderate carbohydrate diet (4.7 g/kg BM/day). Furthermore, James et al. [90] examined the effects of a 3-day high carbohydrate diet (12 g CHO/kg LBM) on muscle glycogen content in endurance trained males and females. Muscle glycogen content significantly increased in response to the high carbohydrate diet in both sexes and during both menstrual cycle phases. Practically, females can carbohydrate load in either phase of the menstrual cycle by adhering to the recommendation of 8-10 g CHO/kg BW in the 3 days leading up to an event. With lower chronic CHO intakes, glycogen storage appears to be more effective with the short term increase in CHO in the luteal phase [25].

Current recommendations call for a carbohydrate intake of 1-4 g/kg BW in the 1–4 h prior to competition or training [84]. Consumption in this timeframe has been shown to increase muscle and liver glycogen and maintain blood glucose, potentially leading to improved performance in the upcoming task [85]. Albeit in trained male cyclists, Coyle et al. [91], determined that a high carbohydrate meal (2 g/kg BW) 4 h prior to an exercise bout resulted in a 42% increase in muscle glycogen content within the vastus lateralis. In several other studies, consumption of a liquid carbohydrate supplement (> 4.5 g/kg BW) at least 3 h before a performance test led to performance improvements in recreational cyclists [92, 93]. While carbohydrate consumption in the hours leading up to an exercise bout has shown beneficial effects in men, data is women is less clear. Pre-exercise carbohydrate feeding is likely to be more important during the follicular phase, when carbohydrate oxidation rates are elevated [25]. Operationally, women participating in aerobic exercise should consume at least 1 g/kg of carbohydrates in the hours leading up to an exercise bout to maximize carbohydrate availability.

The window for carbohydrate consumption does not close at the onset of exercise. Several studies have investigated the effects of carbohydrate supplementation during prolonged endurance exercise specifically over the course of the menstrual cycle. Bailey et al. [94], investigated the ergogenic effects of a 6% carbohydrate solution during a prolonged cycling bout at 70% VO_2_max during both phases of the menstrual cycle. There was a significant increase in TTE in both phases of the menstrual cycle with a dose of 5 ml/kg of the 6% carbohydrate solution versus the placebo. A similar study assessed blood glucose during 120 min of cycling at 70% VO_2_max followed by a time trial, with and without a carbohydrate supplement [7]. During the follicular and luteal phases, supplementing with a 6% carbohydrate solution every 15 min improved performance and significantly minimized differences in blood glucose concentration between menstrual cycle phases [7]. Regardless of menstrual cycle phase, administration of a 6% carbohydrate solution has been shown to improve performance during prolonged endurance exercise. Moreover, consuming 500-1000 mL of the 6% carbohydrate solution falls well within the common guidelines for endurance athletes of 30-60 g/hr. [95].

Following a prolonged endurance exercise bout, replenishing muscle glycogen stores is a top priority [96]. In females, the capacity to restore muscle glycogen stores fluctuates over the course of the menstrual cycle with the highest capacity occurring in the follicular phase [37]. Tarnopolosky et al. [97], investigated the effects of a carbohydrate supplement on muscle glycogen resynthesis following a prolonged bout of submaximal exercise in endurance athletes. All female participants were tested in the follicular phase of the menstrual cycle. Administration of the carbohydrate supplement (0.75 g/kg) immediately following the exercise bout resulted in significantly greater muscle glycogen synthesis compared to the placebo, with no significant sex differences. In addition to satisfying the critical amount of carbohydrates, refueling strategies should also look to minimize the amount of time between the end of exercise and the consumption of carbohydrates. Although performed in males, Ivy [98], determined that delaying carbohydrates in the immediate post-exercise period (2 h) resulted in reduced glycogen storage rates. Therefore, in an ideal situation, females should focus on rapid consumption of at least 0.75 g/kg of carbohydrate following prolonged exercise in order to restore spent muscle glycogen.

**Fig. 3:**
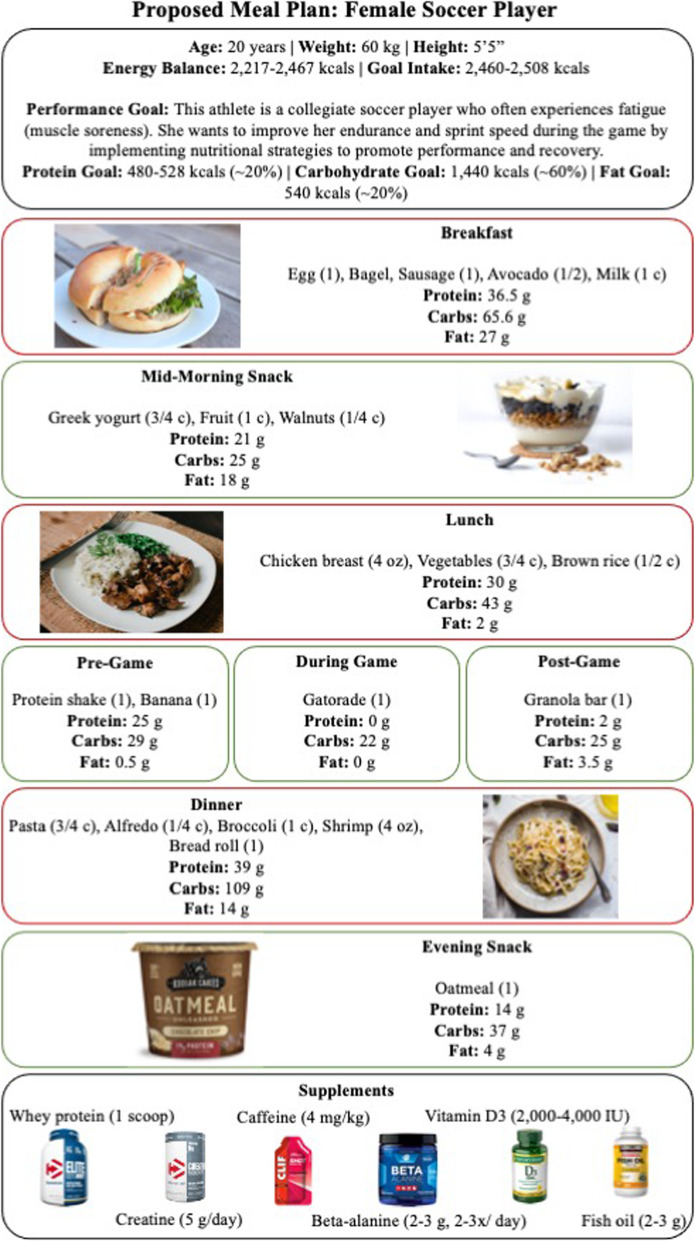
Application for a 20-year-old female who is a soccer player. She is 165.0 cm tall and currently weighs 60 kg. She often experiences fatigue after a game (muscle soreness) and wants to improve her nutritional strategies to enhance performance (sprint speed and endurance). Using the Harris-Benedict equation, her basal metabolic rate (BMR) was determined to be approximately 1430 kcal/day. Considering she is active, her total daily energy expenditure was estimated at approximately 2217–2467 kcal/day by multiplying BMR by an activity coefficient of 1.55–1.725 (one soccer game). A possible macronutrient distribution for performance is 60% carbohydrates (~ 5.6 g/kg), 20% protein (~ 1.9 g/kg), and 20% fat (~ 0.9 g/kg), which was utilized in the proposed meal plan for a game day

While daily carbohydrate needs are determined by length and intensity of activity, practical recommendations about carbohydrate consumption exist for the different time frames surrounding an exercise bout. In preparation for prolonged endurance exercise (> 90 min), females might consider carbohydrate loading by consuming 8-10 g CHO/kg BW in the 3 days leading up to an event, particularly if the event should occur in the follicular phase. In the hours before, 1 g CHO/kg BW should be prioritized to ensure carbohydrate availability throughout the activity. During prolonged endurance exercise, females should consume 500-1000 mL of 6% carbohydrate solution per hour to experience ergogenic effects. Following exercise, females should rapidly consume at least 0.75 g/kg of carbohydrate to begin the process of replenishing muscle glycogen stores. Of course, these recommendations should be considered along with total daily carbohydrate needs. An example for how caloric intake and macronutrients for a female athlete (soccer player) are described in Fig. 3.

## Fats

Fats are essential for maintaining sex hormone concentrations and absorbing fat-soluble vitamins [16]. In females specifically, adequate fat intake may help to sustain normal menstrual cycles [99]. The current acceptable macronutrient distribution range (AMDR) for dietary fat is 20–35% of total calories [82]. In populations seeking a reduction in body fat, recommendations range from 0.5-1 g/kg/day [100]. Additional guidelines are in place for females regarding omega-6 (linoleic acid) and omega-3 (α-linoleic acid) fatty acids, calling for 12 g and 1.1 g per day, respectively [82], a 5–10:1 ratio of omega-6 to omega-3 [101]. Ultimately, females should aim to obtain at least 15% of total calories from unprocessed fat sources like lean meats, nuts, seeds, eggs, and avocados [99].

The variations in female sex hormones during the follicular and luteal phases of the menstrual cycle influence fat metabolism [102]. Elevated estrogen levels during the luteal phase promote lipolysis through increased sensitivity to lipoprotein lipase and increased human growth hormone [103]. During the follicular phase, estrogen levels are lower, resulting in a reduced reliance on fat as an energy substrate [102]. These phenomena persist during submaximal exercise (35–60%) as Hackney et al. [104] demonstrated greater reliance on fat oxidation in the luteal phase versus the follicular phase. Thus, adequate dietary fat intake, particularly during the luteal phase, is essential to account for upregulated fat metabolism.

Previous research has shown that females exhibit lower RER than males during submaximal endurance exercise; therefore, suggesting that females rely more on fat oxidation than males [89, 105, 106]. There also appears to be sex differences in the source of lipids used during submaximal endurance exercise; females used significantly more myocellular triacylglycerol than males during a 90-min bout of cycling at 58% VO_2_max [6]. Consuming a high fat diet (35–57% of energy) following a prolonged bout of endurance exercise restores intramuscular triacylglycerol more effectively than a low-fat diet (10–24%) [107]. Thus, diets lacking adequate amounts of dietary fat can hinder the restoration of intramyocellular lipid stores following endurance exercise, and may negatively influence performance in subsequent exercise bouts [108]. Overall, females must consider adequate dietary fat intake to meet the increased reliance on fat oxidation. From a practical standpoint, females should allocate at least 20% of total energy to dietary fats, not only to meet the demands of sex hormone regulation and fat-soluble vitamin absorption, but also to satisfy sex-specific substrate needs. Additional emphasis should be placed on dietary fat intake during the luteal phase of the menstrual cycle to support the increased reliance on fat metabolism.

## Protein

Dietary protein has many functions in the body with regulation of skeletal muscle mass being a primary role. Skeletal muscle is constantly evolving with a balance between breakdown (muscle protein breakdown [MPB]) and regeneration (muscle protein synthesis [MPS]) [109]. Maintaining adequate protein intake is paramount to ensure that the rate of MPS is at least equal to the rate of MPB, and muscle mass is maintained. Amino acids are critically important since they are the building blocks for new proteins in the body, such as actin in skeletal muscle. Emphasis must be placed on obtaining adequate amounts of essential amino acids, which can only be obtained through food ingestion. Specifically, the essential amino acid leucine has been shown to stimulate MPS through indirect activation of the mammalian target of rapamycin (mTOR). Absence of leucine from the matrix may cause mTOR to enter a refractory period where stimulation via external signals is not possible [110]. The current recommended dietary allowance (RDA) of protein for all sedentary adults over the age of 18 years is 0.8 g/kg/day. This number, however, is likely outdated and is based on the nitrogen balance method, which may not be as accurate as more recent techniques. It has also been suggested that this number is often misunderstood as an optimal level of protein intake rather than a minimal level to prevent muscle loss [111]. ﻿Humayun and colleges [112], found that a better baseline protein intake for sedentary men is likely 1.2 g/kg/day. Additionally, females likely need higher protein intakes due to increased protein oxidation; a baseline starting point for women has been suggested to be ~ 1.6 g/kg/day [113, 114], although more research is needed to fully understand protein requirements for females.

As previously mentioned, female sex hormones (estrogen and progesterone) peak during the mid-luteal phase, which corresponds to an increase in protein oxidation at rest [115, 116]. It has been shown that females require more lysine during the luteal phase than the follicular phase [31]. This is likely due to a number of reasons linked to progesterone upregulation of amino acid use. The spike in progesterone during the mid-luteal phase has been linked to a decrease in amino acid plasma levels, resulting from increased protein biosynthesis from endometrial thickening [116]. Further, protein use during exercise appears to be greater during the mid-luteal phase [117]. Increasing protein consumption is likely warranted during the mid-luteal phase to meet the anabolic demands of the body, especially when exercising.

When combined with resistance training, increased protein intake has a synergistic effect on increases in muscle strength and skeletal muscle mass (i.e. hypertrophy) [109]. For male bodybuilders, it has been shown that the estimated average requirement (EAR) and upper 95% CI RDA (safe intake) for male bodybuilders is ~ 1.7 and ~ 2.2 g/kg/day, respectively [118]. This differs slightly from a study done in female strength athletes that suggests ~ 1.5 g/kg/day and ~ 1.9 g/kg/day for EAR and safe intake respectively [119]. However, a ~ 60% increase from habitual protein intake may be needed to support skeletal muscle hypertrophy [120]. A study of female cyclists and triathletes (endurance athletes) showed that their mean protein requirement was 1.63 g/kg/day, which corresponds with male counterpart recommendations [121]. However, an important consideration of this study is that women were tested during the mid-follicular phase of the menstrual cycle. With increased protein oxidation in the luteal phase, protein needs may be further elevated. Menstrual cycle phase should be considered when evaluating dietary protein needs for females.

Simply meeting protein needs for a given day is important; the timing of protein ingestion must also be considered. A study by Areta and Colleagues [122], examined the influence of 80 g of whey protein isolate supplement (82.9 g of protein) during a 12-hour recovery following resistance exercise. Maximal MPS was achieved through intermediate feeding (20 g every 4 h verses) versus both pulse (10 g every 1.5 h) and bolus (40 g every 6 h) feeding. Other studies have shown that consuming protein prior to exercise may increase muscle protein synthesis compared to post exercise [123]. A study including exclusively women demonstrated that consumption of fat-free milk (a complete animal protein) immediately and 1-h post resistance exercise resulted in significant increases in strength and LM, coupled with a decrease in FM compared to women consuming an isoenergetic carbohydrate [124]. Upper body strength may also be influenced by nutrient timing, as one study found that trained females who consumed a 1:1.5 ratio carbohydrate: protein supplement (16 g CHO: 25 g protein) either pre or post training significantly increased their bench press 1RM following a six week high intensity strength training program compared to no supplement [125]. Interestingly, this same study also demonstrated increased fat oxidation at 30-min post-exercise in the women who consumed the supplement pre-training. Another study by Wingfield and colleagues [126], may offer insight into this increased fat oxidation as they showed that pre-exercise protein ingestion increased REE and decreased RER at both 30- and 60-min following exercise compared to isocaloric CHO consumption. This study also assessed exercise modality and found that high intensity interval training (HIIT) yielded the greatest increase in REE and decrease in RER post-exercise, suggesting that a combination of pre-exercise protein consumption coupled with HIIT may have benefits for body composition and weight reduction in women. Increased dietary protein may also be beneficial when in a caloric or energy deficit (e.g. weight loss/body recomposition). A recent review paper reported that high levels of dietary protein in women supports the maintenance of muscle mass and whole-body protein homeostasis during caloric deficit, but the beneficial effects of a high protein diet are decreased as the deficit increases [127]. Additionally, while in a caloric deficit, women who consumed more of their calories from PRO compared to CHO demonstrated an increase in the fat/ lean mass loss ratio resulting in a greater percentage of weight loss from FM while sparing LM [10].

**Fig. 4:**
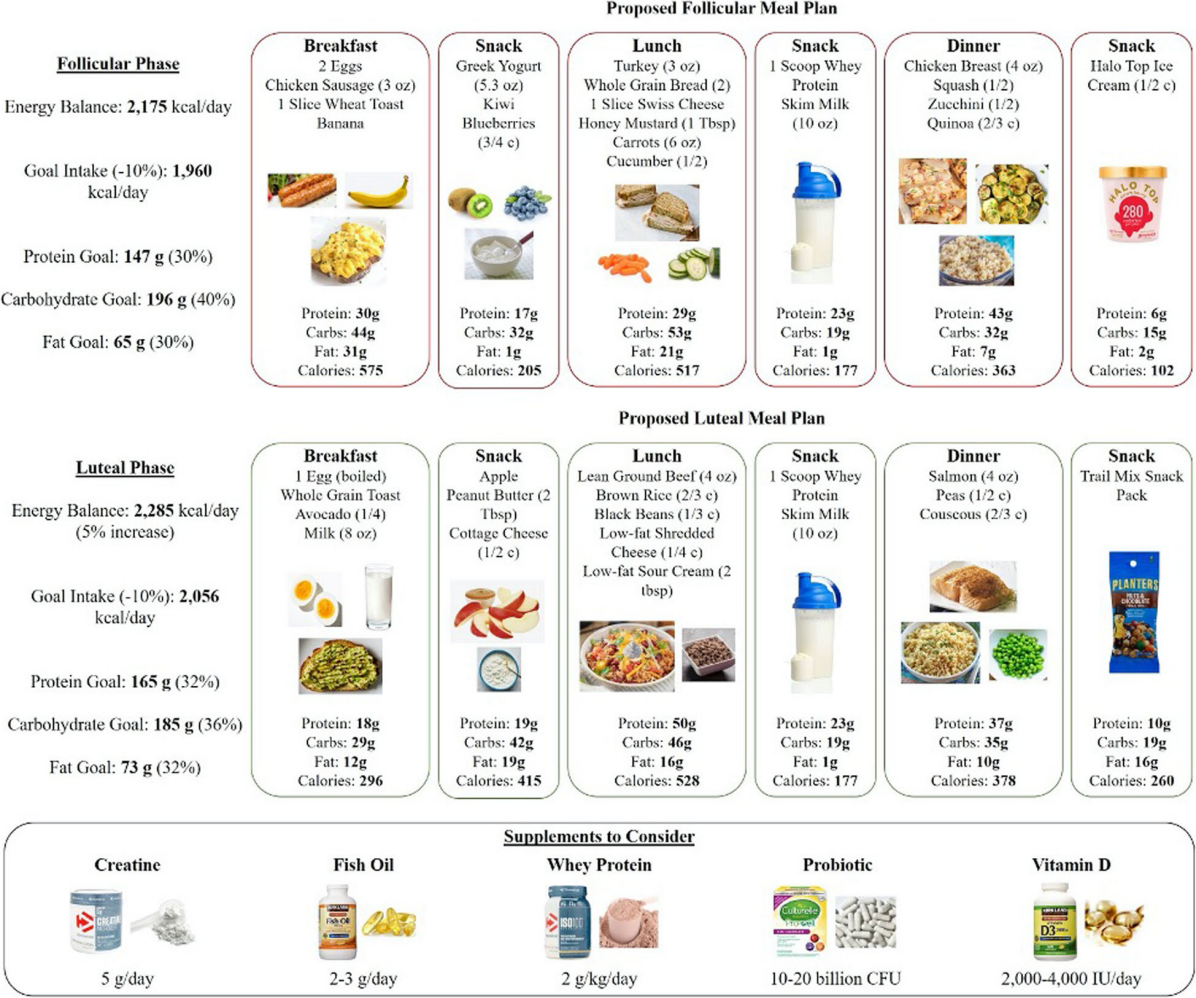
Application for a 38-year-old female whose primary goal is weight loss. She is 161.5 cm tall and currently weighs 86.4 kg, which places her in the obese category based on a BMI of 33 kg/m^2^. Her current goal weight is 77 kg, which would ultimately reduce her BMI to 29.6 (overweight). Using the Harris-Benedict equation, her basal metabolic rate (BMR) was determined to be approximately 1580 kcal/day. Considering she is moderately active; her total daily energy expenditure was approximately 2175 kcal/day by multiplying BMR by an activity coefficient of 1.375. Because her goal is weight loss, her calorie intake has been reduced by 10% to ultimately reach a hypocaloric intake of 1960 kcal/day during her follicular phase. Because energy expenditure is increased by 2.5–11% in the luteal phase, her energy balance requirement of 2175 kcal/day was increased by 5% for a baseline intake of 2285 kcal/day. Similarly, this value was reduced by 10% (2056 kcal/day) in order to ensure a caloric deficit for weight loss. A potential macronutrient distribution for weight loss was set at 40% carbohydrates (~ 2.4 g/kg), 30% protein (~ 1.8 g/kg), and 30% fat (~ 0.8 g/kg), which was utilized in designing the follicular proposed meal plan. As a result of increased protein and fat oxidation during the luteal phase, which is accompanied by a desire to eat saltier and sweeter foods, this distribution was altered slightly to 36% carbohydrates, 32% protein, and 32% fat in the luteal phase [128]

Evidence suggests that females have increased daily protein needs well above the current RDA of 0.8 g/kg/day. Female strength and endurance athletes should consume a minimum of 1.6 g/kg/day. If hypertrophy is the goal, an additional 60% deviation from their habitual intake may be needed [120]. Spacing protein consumption throughout the day in 20–30-g servings to meet the daily requirement is more optimal compared to than one large bolus or smaller and frequent feedings. For weight loss, it is recommended that protein consumption be increased even further to ensure the majority of weight loss is FM, while LM is maintained. Resistance exercise may have a synergistic effect of maintaining muscle mass with a high protein diet during times of caloric deficit; as such females may consider implementing a resistance-based training program. Females should also consider cycle phase as there may be an increased demand for protein during the luteal phase. An example of how a woman may consider menstrual cycle phase into caloric and macronutrient needs is depicted in Fig. 4.

## Dietary supplements

The majority of dietary supplements have been evaluated primarily in men; based on physiological theory and sex-physiology, the following sections introduce potential dietary supplements that may be efficacious for women. Table 1 outlines each supplement, potential implication for taking the supplement, and the dosage needed to be effective. Although there does not appear to be major sex-differences in the recommendations for dosages or usage of key dietary supplements; this summary aims to highlight supplements that have been studied in women and have been shown to be efficacious. Additionally, some of the supplements that have been discussed here appear to have a differential mechanistic effect (i.e. creatine) or notable points (i.e. half-life, timing, etc), for example. We must also recognize that the evaluation of dietary supplements in women needs additional attention; this section highlights ingredients that may be of particularly importance for active women.

**Table 1:** Dietary supplements that may be beneficial for women

Supplement	Purpose	Dose
Beta-alanine	Enhance exercise performance	4-6g/day in 1-2g divided doses for 4 wks
	Acts as a buffer	A slower releasing supplement can increase skeletal muscle carnosine levels more than a rapid releasing supplement
	Fatigue attenuation	
	Optimize recovery	
Caffeine	Increase athletic performance	Doses between 3-9 mg/kg/ allow ergogenic effects when consumed 60 minutes before exercise
	Spare glycogen by increasing fat metabolism	
	Decrease pain perception	
Calcium	Vital to muscle contraction and relaxation	1000mg/day
	Helpful for bone density	
Creatine Monohydrate	Enhance exercise performance	0.3g/kg of body weight 4 times a day for 5 to 7 days for a “loading phase”
	Decrease injury risk	
	Enhance rehabilitation	
	Decrease disease risk in young, middle aged, and old age groups	
	Increased fat free mass	Followed by a "maintenance" phase where one where one should ingest 5g/day
	Effective during menses, pregnancy, and post-partum (regulate mood and decrease depression)	
	Beneficial to mental health	
	Beneficial to bone health	
	Neuroprotection	
Folate	Decrease chance of anemia	400ug/day
Iron	Decrease chance of anemia	18mg/day
Omega-3	Anti-inflammation	To see the benefits of omega-3, 1-3g should be consumed a day
	Decrease the overall risk of disease	
	Plays a vital role in growth and development	
	Improve immune function	
	Decrease depression	
Probiotics	Meet overall health needs	Consuming a multistrain probiotic
	Regulate immune and digestive function	Probiotics taken daily should include 10 to 20 billion colony forming units (CFU)
	Regulate uro-genital tract	
	Promote skin health	
	Reduce inflammation	
Riboflavin	Help skin	1.1mg/day
	Regulate endocrine function	1.6mg per 1000 calories (if exercising or lactating)
Vitamin B12	Decrease chance of anemia	2.4ug/day
Vitamin D3	Promote bone health	2,000 to 4,000 IU is safe and beneficial
Whey Protein	20-30g boluses throughout the day to reach the recommended 1.6g/kg/day for active women	20-30g boluses throughout the day to reach the recommended 1.6g/kg/day for active women
	Improve body composition	
	Increase bone mineral density	
	Important to muscle protein synthesis	
	Increase time to exhaustion during exercise	
	Improve recovery	

### Beta-alanine

Beta-alanine is a non-essential amino acid that enhances exercise performance through increasing muscle carnosine levels and acts as a hydrogen ion buffer, thereby lowering pH [129]. Increasing muscle carnosine has resulted in improvements in exercise performance lasting primarily 2–4 min [129]. While the majority of data with beta-alanine supplementation is in males, women have reported lower initial muscle carnosine levels, suggesting they could potentially see greater benefits compared to men [130]. Varanoske et al. [131] reported 28 days of beta-alanine supplementation was more effective for women, than men, for increasing muscle carnosine, although fatigue attenuation was similar for both men and women [131]. In a later study, Varanoske et al. [132] examined the influence of muscle carnosine levels of fatigue during resistance training and discovered that women who consumed more dietary protein had higher carnosine levels, those with higher carnosine levels, were able to delay fatigue longer than the women with lower carnosine levels. In a more recent paper [133], when selecting slow releasing or rapid releasing beta-alanine supplements, a slower releasing beta-alanine supplement taken 6 g per day for 28 days increased skeletal muscle carnosine levels by 16.4% more than consuming a rapid releasing supplement [133]. Individuals who suffer from low muscle carnosine levels such as older adults, women, and vegetarians could benefit from supplementing with beta-alanine [134]. Beta-alanine supplementation recommendations should not differ between men and women. Harris and company [135] showed that a total dose of 4–6 g/day divided into 1–2 g doses for 4 weeks increases muscle carnosine concentrations by 64%. Overall, beta-alanine may be effective for delaying fatigue and/or optimizing recovery in women. Of note, beta-alanine often results in a paresthesia or tingling side effect; this is harmless, and may actually be more prevalent in males vs. females [136].

### Caffeine

Caffeine is a popular, natural ergogenic aid that causes physiological response by acting upon adenosine receptors and acts as a central nervous system stimulant [137], please see ISSN Position Statement for extensive details on caffeine [138]. Caffeine elimination fluctuates over the course of the menstrual cycle, and some women feel the effects of caffeine longer during their luteal phase [139]. Previous literature shows women can accumulate caffeine during the luteal phase prior to beginning menstruation, and experience the effects of caffeine longer [139]. These effects can increase premenstrual symptoms in some women, as well as intensify normal effects of caffeine such as cardiovascular effects (i.e. heart rate), anxiety, and impaired sleep [139–141]. During anaerobic and aerobic exercise, caffeine can act as a ergogenic aid [142]. A resistance training study in women showed that caffeine (6 mg/kg) is effective for increasing strength performance and increasing bench press 1RM [143]. Caffeine has also been shown to effective for aerobic exercise performance as it spares muscle glycogen by increasing fat metabolism [142]. Caffeine has also been known to decrease pain perception which would be helpful prior to all types of exercise [142]. Doses between 3 and 9 mg/kg allow ergogenic effects when consumed 60 min before exercise [142]. Little evidence exists that would suggest a need for a different dose in women.

### Creatine monohydrate

Previous studies have shown that creatine supplementation can improve athletic performance, decrease injury risk, enhance rehabilitation, and decrease disease risk in young, middle aged, and old age groups. Creatine works by increasing muscle phosphocreatine stores, which can increase energy availability [144, 145] (see ISSN position stand on creatine: [146]). When discussing nutrition for women, the menstrual cycle must be taken into consideration as hormonal changes occur across the cycle. Several studies acknowledge the gap in the literature involving female’s supplementation with creatine and have found that creatine can be effective during menses, pregnancy, and post-partum. Also, as women age, creatine has been found to be beneficial for improving mental health, bone health, and physical function [147]. Although many studies highlight creatine’s ability to enhance performance, creatine is also beneficial to overall health, specifically brain health. Creatine can help cognitive abilities, regulate mood, decrease depression, and offer neuroprotection, particularly in women [148]. Recent literature has shown that creatine supplementation can protect the brain from traumatic brain injuries (TBI) and help recover from TBI [148].

Although men appear to be more sensitive to creatine supplementation, improved athletic performance and increased fat free mass have been observed in both sexes [147, 149, 150]. Moreover, in a study looking at high intensity interval training (HIIT), males and females who consumed creatine monohydrate had increased peak and relative peak anaerobic cycling power, dorsiflexion maximal voluntary contraction (MVC) torque, and increased lactate [151]. Creatine supplementation also has been shown to improve mean strength and endurance during repeated contractions in women [152]. There are two effective strategies to increase creatine stores; 1) a loading phase which requires ingestion of 0.3 g/kg of body weight four times a day for 5 to 7 days followed by a “maintenance” phase of 3–5 g/day, or 2) a daily 5 g dose. Note this dosing strategy takes longer to increase Cr stores [149, 153]. These dosing strategies will allow for an increase in muscle creatine stores as well as the previously mentioned health benefits. One potential side effect from creatine supplementation is related to weight gain, related to an increase in total body water. Weight gain with creatine supplementation is more prevalent in men; for women, weight gain may be more likely during the luteal phase due to hormonally related fluid shifts [154]. However, this is likely to occur only with a loading dose.

### Omega 3

Omega-3 plays a role in anti-inflammation within the body and has been shown to decrease the risk of disease [155]. The two most active eicosanoids derived from omega-3 are DHA (docosahexaenoic acid) and EPA (eicosapentaenoic acid). DHA and EPA play a vital role in growth and development as well as decreasing cytokines within the body and improving immune function [155]. Previous literature has shown that individuals who suffer from rheumatoid arthritis, inflammatory bowel disease, or asthma can see improvements in symptoms from supplementing with EPA and DHA [155]. Omega-3 is not only important for physical health, but also mental health as evidence shows that individuals who consume more omega-3 are less likely to be depressed [156]. Omega-3 fatty acids aid in growth and development as they are often supplemented during pregnancy with previous literature showing benefits for both the mother and infant [157]. Essential fats are also needed to counteract the Triad and RED-S, which were discussed previously. Women who over train and under consume calories can experience detrimental side effects, such as amenorrhea and loss of BMD [99]. Omega-3 supplementation may help to address the higher inflammatory response seen in women following exercise; increased omega-3 levels have also been shown to reduce symptoms of depression and anxiety, particularly in women. To see the benefits of omega-3, 1-3 g daily should be consumed [158].

### Probiotics

Probiotics have become a popular dietary supplement [153]. They have been shown to improve the bacterial composition within the intestines, regulate immune and digestive function, and aid uro-genital tract and skin health [159] (see ISSN position statement for extended discussion: [160]). Previous research states that probiotic supplementation can improve intestinal function and reduce inflammation [161]. Probiotics can even be helpful in treating recurring urinary tract infections (UTIs) in some women [162]. A recent study relevant to females, demonstrated an improvement in iron levels and absorption when 20 mg of iron (ferrous-fumarate) was combined with *lactobaccilus plantarum* 299v (LP299V, 10^10^ CFU), suggesting this probiotic strain may help improve iron levels [163]. Probiotics should be chosen based on strain and desired outcome; strains may vary for women compared to men [164, 165]. Consuming a multistrain probiotic supplement may be the most feasible way to experience these health benefits; additionally, being strategic in choosing a single strain would be beneficial based on the reported function of the specific strain. The probiotic supplement should be taken daily and should include 10 to 20 billion colony forming units (CFU) [166], or a clinically validated strain at effect doses [160].

### Protein supplementation

Women could greatly benefit from protein supplementation to meet their daily protein needs, especially during the luteal phase of the menstrual cycle, which is marked by increased protein oxidation [113, 114]. There are several popular protein supplements with evidence to support beneficial effects. These include collagen peptides, essential amino acids, plant-based proteins, and whey. *Collagen peptides*, when paired with resistance training, can improve body composition and increase muscle strength [167]. Additionally, collagen peptide supplementation increases bone formation while reducing bone degradation in postmenopausal women. This increase bone mineral density has been shown with a dosage of 5 g/day for one year [158]. It must be recognized that collagen protein is not a complete protein. This suggests that it is often more effective to consume a collagen protein with a complete protein (i.e. whey, EAA, dairy). *Essential amino acids* (EAAs) are important to muscle protein synthesis and can have ergogenic effects [168]. Previous literature has shown that six weeks of EAA supplementation (18.3 g/day) improves TTE, a measure of aerobic muscular endurance [168]. Additionally, consuming 6–12 g of EAA alone or in part of a protein supplement of 20–40 g can stimulate muscle protein synthesis [169]. *Plant-based proteins* have also become popular sources of protein supplementation and are usually seen as legume, nut, or soy protein [170]. Because of the amino acid profiles of the different plant-based sources, multiple types must be combined, or the addition of single amino acids, in order to adequately stimulate MPS [170, 171]. If consuming a plant-based protein, it is recommended to potentially increase the serving size or a probiotic as a way to enhance the amino acid absorption [172]. This technique ensures that enough leucine is consumed to stimulate MPS. Whey protein is the highest quality form of protein and is available as hydrolysate, isolate, and concentrate. Whey protein isolate is pure with protein concentration > 90% due to lactose and fat being removed [170] and may be most beneficial for women to prevent gastrointestinal distress. Previous data has supported the use of protein prior to and/or after exercise in women to enhance muscle recovery and LM [173].

### Vitamins and minerals

The addition of supplemental vitamins and minerals can benefit highly active individuals. Female athletes that are menstruating may have an increased need for certain vitamins and minerals. Specifically, female athletes are often lacking in folate, riboflavin, and B12 [16]. Deficiencies in folate and B12 can cause anemia, which hampers athletic performance [174]. The average folate intake in women is 126–364 μg/day. This measure is well below the current RDA of 400 μg/day [16]. Folate supplementation is a simple, effective method to meet current recommendations and avoid performance decrements. The RDA for Riboflavin is 1.1 mg/day for women. Notably, this standard was established in 1998 [175] and may not reflect emerging research. Those deficient in Riboflavin can experience sore throats, hyperemia, swelling of the mouth and throat, and skin disorders [175, 176]. Riboflavin is usually 1.4 mg per 1000 cal, but women exercising or lactating should consume 1.6 mg per 1000 cal [16, 175]. The RDA for B12 for adults is 2.4μg/day [175]. There are little known differences between men and women, but it is important for women who are pregnant and nursing to consume adequate doses of B12 to give to their child. Individuals following plant-based diets usually lack B12 as it is most commonly consumed from meat and the bioavailability is low in plants [177]. As mentioned previously, women who are lacking B12 can become anemic and feel fatigued faster when exercising [175]. In addition to lacking B vitamins, some individuals lack vitamin D as well. The RDA for Vitamin D is 600 IU for both men and women 9 to 70 years of age. Individuals over 70 years of age should increase consumption to 800 IU [178]. Previous literature has shown that dosages of 2000 to 4000 IU are safe and beneficial [178]. Vitamin D closely works with calcium to promote bone health. Those deficient in vitamin D can have poor mineralization of the bone and lead to skeletal disorders, especially as one ages [178].

Female athletes have lower calcium intakes when compared to their male counterparts. Additionally, those with dairy sensitivities are at an even greater risk for low calcium levels [16]. The daily recommended intake (DRI) of calcium for adult females is 1000 mg/day [178]. Supplementation is a viable alternative for female athletes who do not consume dairy or who are under consuming calories [16]. Calcium is also vital to muscle contraction and relaxation; therefore, supplementation may promote optimal muscle function [178]. Iron deficiencies are extremely common among female athletes especially those that are vegan/vegetarian and under consuming calories. The ISSN recommends males consume 8 mg/day, while women should consume 18 mg/day [153].

## Conclusions and practical applications

Emerging evidence underscores the importance of sex specific nutritional strategies and recommendations for females, particularly active females. Key physiological differences between the sexes are brought about by differences in sex hormone concentrations; however, intra-individual differences also exist in females, throughout the menstrual cycle and throughout the lifecycle (i.e. puberty, pregnancy, menopause). These differences occur during phases of the menstrual cycle, which are due to fluctuating hormonal levels, for example increased estrogen and progesterone during the mid-luteal phase [25]. Because of this, females may benefit from separate sex specific nutritional recommendations, especially when engaging in regular exercise. Specific caloric, macronutrient, micronutrient, and supplement recommendations should be tailored to the individual to meet their desired goals, but basic requirements and staring points are likely universal, and thus addressed within this review. Further, timing and dosing must be considered, especially when performance or recovery are primary goals. These distinct nutritional guidelines and recommendations for females are warranted given the sex-based difference but have been lacking to date. There is also a significant lack of studies assessing female specific nutritional strategies for health, performance, and body composition. Future additional research evaluating female specific nutritional strategies is needed, especially for active women.

## Data Availability

Not applicable.

## Abbreviations

%fat: Body fat percentage
1RM: One repetition maximum
AMDR: Acceptable macronutrient distribution range
BMD: Bone mineral density
CK: Creatine kinase
DHA: Docosahexaenoic acid
EAA: Essential amino acids
EAR: Estimated average requirement
EPA: Eicosapentaenoic acid
FFA: Free fatty acids
FM: Fat mass
FSH: Follicle stimulating hormone
HIIT: High intensity interval training
IL-6: Interleukin-6
LH: Luteinizing hormone
LM: Lean mass
MPB: Muscle protein breakdown
MPS: Muscle protein synthesis
mTOR: Mammalian target of rapamycin
MVC: Maximal voluntary contraction
RDA: Recommended dietary allowance
RED-S: Relative energy deficiency in sport
REE: Resting energy expenditure
RER: Respiratory exchange ratio
TBI: Traumatic brain injury
TEE: Time to exhaustion
Triad: Female athlete triad
